# A Digital Mental Health Intervention (Inuka) for Common Mental Health Disorders in Zimbabwean Adults in Response to the COVID-19 Pandemic: Feasibility and Acceptability Pilot Study

**DOI:** 10.2196/37968

**Published:** 2022-10-07

**Authors:** Jermaine Dambi, Clara Norman, Asmae Doukani, Stephan Potgieter, Jean Turner, Rosemary Musesengwa, Ruth Verhey, Dixon Chibanda

**Affiliations:** 1 Rehabilitation Sciences Unit Faculty of Medicine and Health Sciences University of Zimbabwe Harare Zimbabwe; 2 Friendship Bench Harare Zimbabwe; 3 Research Support Centre Faculty of Medicine and Health Sciences University of Zimbabwe Harare Zimbabwe; 4 Department of Population Health Faculty of Epidemiology and Population Health London School of Hygiene and Tropical Medicine London United Kingdom; 5 Inuka Foundation Almere Netherlands; 6 Department of Psychiatry University of Oxford London United Kingdom

**Keywords:** acceptability, COVID-19, feasibility, Friendship Bench, Inuka, pilot, task-shifting, Zimbabwe

## Abstract

**Background:**

Common mental health disorders (CMDs) are leading causes of disability globally. The ongoing COVID-19 pandemic has further exacerbated the burden of CMDs. COVID-19 containment measures, including lockdowns, have disrupted access to in-person mental health care. It is therefore imperative to explore the utility of digital mental health interventions to bridge the treatment gap. Mobile health technologies are effective tools for increasing access to treatment at a lower cost. This study explores the utility of Inuka, a chat-based app hinged on the Friendship Bench problem-solving therapy intervention. The Inuka app offers double anonymity, and clients can book or cancel a session at their convenience. Inuka services can be accessed either through a mobile app or the web.

**Objective:**

We aimed to explore the feasibility of conducting a future clinical trial. Additionally, we evaluated the feasibility, acceptability, appropriateness, scalability, and preliminary effectiveness of Inuka.

**Methods:**

Data were collected using concurrent mixed methods. We used a pragmatic quasiexperimental design to compare the feasibility, acceptability, and preliminary clinical effectiveness of Inuka (experimental group) and WhatsApp chat-based counseling (control). Participants received 6 problem-solving therapy sessions delivered by lay counselors. A reduction in CMDs was the primary clinical outcome. The secondary outcomes were health-related quality of life (HRQoL), disability and functioning, and social support. Quantitative outcomes were analyzed using descriptive and bivariate statistics. Finally, we used administrative data and semistructured interviews to gather data on acceptability and feasibility; this was analyzed using thematic analysis.

**Results:**

Altogether, 258 participants were screened over 6 months, with 202 assessed for eligibility, and 176 participants were included in the study (recruitment ratio of 29 participants/month). The participants’ mean age was 24.4 (SD 5.3) years, and most participants were female and had tertiary education. The mean daily smartphone usage was 8 (SD 3.5) hours. Eighty-three users signed up and completed at least one session. The average completion rate was 3 out of 4 sessions. Inuka was deemed feasible and acceptable in the local context, with connectivity challenges, app instability, expensive mobile data, and power outages cited as potential barriers to scale up. Generally, there was a decline in CMDs (*F*_2,73_=2.63; *P*=.08), depression (*F*_2,73_=7.67; *P*<.001), and anxiety (*F*_2,73_=2.95; *P*=.06) and a corresponding increase in HRQoL (*F*_2,73_=7.287; *P*<.001) in both groups.

**Conclusions:**

Study outcomes showed that it is feasible to run a future large-scale randomized clinical trial (RCT) and lend support to the feasibility and acceptability of Inuka, including evidence of preliminary effectiveness. The app’s double anonymity and structured support were the most salient features. There is a great need for iterative app updates before scaling up. Finally, a large-scale hybrid RCT with a longer follow-up to evaluate the clinical implementation and cost-effectiveness of the app is needed.

## Introduction

Common mental health disorders (CMDs) are leading causes of disability-adjusted life years, with more than a billion people having mental disorders globally [1,2]. The burden of CMDs is disproportionately large in low-resourced settings, with 1 in every 4 people at risk of having a CMD in a lifetime [3]. Globally, the burden of mental disorders has invariably increased due to the ongoing COVID-19 pandemic [4]. The absence of social safety nets, and the presence of poverty, food insecurity, impairment of social and occupational functioning (eg, loss of income and recreation opportunities), and uncertainty have predisposed the Zimbabwean population to poor mental health as in other low-resourced settings [5,6]. The World Bank projects a global recession after the COVID-19 pandemic [7]. The economic effects could further shrink the Zimbabwean economy, predisposing the majority of the population to greater poverty [5]. Poverty has been linked to poor mental health functioning, thus creating a vicious cycle [7]. Unfortunately, if untreated, CMDs may lead to poor daily functioning, economic losses due to lost productivity at work, risky sexual behaviors, substance misuse, relationship instability, suicidal ideation, and increased health care resource use, which can further pressure fragile health care systems [8-10].

Despite the high burden of CMDs, a vast gap in mental health care exists in low-resourced settings. The general population is unlikely to seek mental health services due to various environmental and personal factors [8,10-12]. First, the stigmatization of mental health services is a salient barrier to mental health seeking and use behaviors [10-13]. Second, the general population may not inherently recognize the need for mental health care [10,11]. For example, due to low mental health literacy, the general population may perceive CMD symptoms as normal pressure associated with adulthood and thus may not actively seek treatment [10,11]. Third, not all communities have functional mechanisms for the early screening, detection, referral, and treatment of CMDs [11]. Like other low-income countries, mental health care has not been prioritized in Zimbabwe, leading to a substantial care gap further exacerbated by COVID-19 [14]. Fourth, numerous competing health and social needs (eg, COVID-19, HIV/AIDS, and maternal and child health programs) coupled with a shortage of trained mental health professionals (eg, clinical psychologists, occupational therapists, and psychiatrists) are salient barriers to the provision of mental health services [14,15]. Considering these inhibitive factors, it is important to explore alternative ways to provide mental health services to the general population, which address public stigma and increase access at a lower cost.

The Friendship Bench, a task shifting–based intervention, evolved as an innovation to reduce the mental health care gap in resource-constrained settings [14]. Task shifting entails redistributing less complicated tasks to less-qualified cadres to increase care coverage and mitigate human resource gaps [16]. For instance, in the absence of trained mental health care practitioners, the World Health Organization (WHO) recommends the assignment of lay counselors to provide mental health care services under the supervision of higher-level cadres (eg, psychiatrists) [16]. The Friendship Bench uses lay counselors who are primarily community volunteers without expert training in health care; they are trained to provide basic health promotion, including immunizations [14,15]. Trained lay counselors provide mental health services using 6 sessions of problem-solving therapy (PST). The Friendship Bench intervention is a proven evidence-based intervention found to reduce CMDs among adults attending primary health care clinics and has been adopted internationally and as a Zimbabwean national treatment/care model [14,15]. As part of efforts to scale up the intervention, the Friendship Bench is exploring the utility of multiple modal intervention delivery methods. For example, Friendship Bench uses the WhatsApp platform and telephone calls to deliver therapy sessions. Exploring alternatives to physical (in-person) psychosocial interventions is paramount, given the ongoing COVID-19 pandemic. The impending public health catastrophe requires a proactive approach to mental health service provision.

Systematic reviews have demonstrated the effectiveness of mobile- and web-based applications for treating CMDs in the general population [9,10,13,17]. Zimbabwe has recently seen an exponential increase in digital penetration. The general population has access to digital devices (eg, smartphones, tablet devices, and personal laptops) and the internet, and up to 90% of Zimbabweans own a smartphone [7]. High digital penetration is an essential predictor of the utility of digital mental health services [9-11,13,17]. Considering the potential reach of digital mental health interventions, it is vital to identify and evaluate the feasibility and scalability of mobile health (mHealth) technologies, given resource limitations in low-to-middle income countries. Inuka, a digital-based mental well-being service, is potentially low-hanging fruit for increasing mental health care coverage. The Inuka app transferred the Friendship Bench intervention to a digital setting to make the service more accessible and scalable [18]. Chat-based mental health care services have been found to lower barriers to seeking support from the general population and increase active/effective help-seeking behavior [12]. Like physical Friendship Bench sessions, in Inuka, therapy is provided by lay counselors using PST. Unlike physical Friendship Bench sessions, in Inuka, clients can book or cancel appointments at their convenience. Further, the Inuka app requires low bandwidth, offers prospective users complete anonymity, and has been tested in Kenya, a country with an almost similar sociodemographic profile to Zimbabwe [18]. In the Kenyan pilot cohort study (N=60), participants showed significantly declined psychiatric morbidity after 4 sessions [18]. Further, most participants perceived the Inuka app to be very helpful in dealing with stressful conditions (n=38, 69%), reported that sessions met their needs (n=27, 49%), and rated the program as excellent (n=36, 66%) [18]. Although preliminary evidence is promising, there is a need to explore the multilevel factors influencing the feasibility of implementing Inuka in a different context [19]. For instance, connectivity challenges, power outages, and local perceptions of mental health interventions may affect the uptake, feasibility, and acceptability of mHealth solutions in Zimbabwe. Moreover, the Kenyan pilot study was clinic based, limiting our understanding of the implementability of the services outside primary health care settings. It is also vital to glean data on users’ experiences to inform the digital intervention’s feasibility and acceptability, using qualitative and quantitative methods. Finally, there is a great need to evaluate the effectiveness of mHealth interventions in low-resource settings using randomized clinical trials (RCTs) [20]. However, mHealth interventions are still in infancy in low-resource settings, and pilot studies are required to assess the feasibility of conducting future clinical trials. Therefore, the study sought to address the following objectives: (1) to assess the feasibility of a large-scale clinical trial by exploring the feasibility of randomization, recruitment rate, and retention rate; (2) to explore the feasibility, acceptability, appropriateness, and scalability of the Inuka intervention for providing mental health care in the Zimbabwean general population; (3) to evaluate the preliminary clinical effectiveness of the Inuka app for improving the general population’s mental health compared with the WhatsApp intervention.

## Methods

### Design

Concurrent mixed methods were used to simultaneously glean data on implementation-related parameters (feasibility and acceptability) and preliminary clinical effectiveness [21]. A pragmatic nonequivalent control group quasiexperimental design [22] was used, with the Inuka intervention arm being the experimental group and the WhatsApp intervention arm being the control group. In line with the pragmatic design adopted, participants were assigned to either Inuka or WhatsApp PST counseling sessions, depending on preference and bandwidth/connectivity stability. In response to physical/social distancing measures to mitigate the effects of the COVID-19 pandemic, this study aims to pilot test digital Friendship Bench interventions (Inuka and WhatsApp) in the general population under realistic conditions. We want to understand how the 2 interventions may best be implemented. The “classical/in-person” Friendship Bench intervention has demonstrated effectiveness. It was assumed that both Inuka and WhatsApp counseling would be equally effective in improving the mental health of the general population. Semistructured interviews were used to gather data on aspects of implementation.

### Participants

The inclusion criteria for participation were as follows: age of 18 years or older; score of 9 or more on the Shona Symptoms Questionnaire (SSQ), a locally validated screening tool for CMDs with good psychometric properties [14]; and access to a smartphone/tablet or a laptop/desktop computer. People were excluded from the study if they were receiving specialist mental health care at the point of recruitment and had known psychiatric conditions such as major depression.

### Interventions

There were 2 treatment arms. The experimental intervention consisted of the Inuka intervention, a digital chat-based mental health application [18]. The control intervention consisted of the Friendship Bench WhatsApp intervention (see Multimedia Appendix 1 for more information on the Inuka intervention). Both interventions consisted of 6 PST sessions. Session 1 involves identifying possible stressors and ranking them in the order of priority. Sessions 2 to 5 involve developing context-specific and need-driven solutions. Finally, session 6 involves reviewing the client’s progress. Interventions across both arms were delivered by nonspecialist lay counselors/peers trained to deliver PST. Lay counselors consisted of young adults aged 18 to 25 years. Sessions across both interventions were chat based. However, those allocated to the control arm also had an option of voice notes on WhatsApp. For quality assurances, trained clinical psychologists and psychiatrists supervised lay counselors from both arms to ensure fidelity to the therapy/intervention.

### Procedure

We advertised the pilot study through the Friendship Bench website [23] and social media (ie, Facebook, WhatsApp, Instagram, and Twitter). Prospective participants filled out a sign-up form by entering their email address and phone number or sending a text or WhatsApp message to the Friendship Bench customer care number. All contact details were forwarded to the principal investigator (PI) to initiate contact with potential participants. Two research assistants (RAs) followed up with all prospective participants within 30 minutes of receiving an inquiry through a phone call. The RAs briefly described the study and assessed clients for eligibility. Eligible participants were then sent consent forms, and return was expected within 48 hours. The RAs followed up with nonresponders 48 hours from when consent forms were sent out. After consenting to participation, clients were given the option to choose the Inuka or WhatsApp intervention depending on their preference and the stability of the internet connection. Participants choosing WhatsApp were asked their preferred date and time for their first session. The preferred times were relayed to the Friendship Bench customer care unit. Bookings were only finalized after cross-checking with the client.

For participants choosing Inuka, the RAs briefly explained the sign-up process and thereafter sent the client a registration link and step-by-step guide for the signing-up process. Participants could either sign-up independently or be assisted by the RAs. For Inuka, clients only enter their phone number and email address. The system creates a pseudonym to enable anonymity. The phone number is captured for follow-ups, with only the administrators accessing the client credentials. Both the client and the counselor are blinded to the other entities’ phone numbers, and communication is only done through the app. Clients can, however, send offline messages between sessions. Upon signing up, the system sends a client an email confirming the date, time, and name of the counselor; the session calendar file; and a link to download the app. Participants were encouraged to download and install the app, and sign in using their sign-up details. Inuka can either be accessed through a browser or the app. The app is currently available for Android users only; an iOS app version is under development. Upon consulting various stakeholders (clinicians, lay counselors, service users, and app developers), it was deemed essential to first develop the Android version of the Inuka app. The Android platform dominates the smartphone market in low-income countries. From the administrative back end, the PI had access to the list of all clients who had signed up. The list was shared with RAs who followed up with clients to book an appointment to collect baseline measures. Please refer to Multimedia Appendix 1 for a graphic illustration of the Inuka app. Finally, all baseline data collection was performed before the first session across the groups.

### Outcomes

#### Future Clinical Trial Feasibility

To measure the feasibility of a future clinical trial, we collected data on the following feasibility indicators: (1) willingness to be randomized, (2) recruitment rate and source, (3) recruitment fidelity, (4) eligibility and consent rates, and (5) attrition rates.

#### Implementation Parameters

We used the Consolidated Framework for Implementation Research (CFIR) [24] to identify and conceptualize the implementation parameters. We assessed the feasibility, acceptability, appropriateness, adoption/uptake, and fidelity of Inuka services. Additionally, we used the mHealth App Usability Questionnaire (MAUQ) to measure lay counselors’ satisfaction with the Inuka app. The MAUQ is a psychometrically robust 20-item tool for assessing user experiences with mHealth technologies [25]. User experiences and perceptions are evaluated under 4 domains (ie, engagement/interactivity, ease of use, esthetics, and information quality). Items are scored on a 5-point Likert scale, giving scores in the range of 20 to 100, with higher scores indicating greater usability and usefulness [25]. Table 1 outlines the definitions and operationalization of implementation indicators, including the evaluation methodology.

**Table 1:** Implementation parameters.

Domain	Working definition	Operationalization/indicators	Methods	Stage
Feasibility of implementation	The extent to which an innovation (Inuka) can be successfully used to provide mental health services in the Zimbabwean general population.	● Patients’ experiences with the Inuka platform (ie, ease of use of the platform). ● Administrators’ and lay counselors’ experiences in rolling out the digital app.	● Semistructured interviews with the general population, lay counselors, and administrators ● Mobile Application Rating Scale (MAUQ) scores [25] ● Administrative data	Postimplementation
Acceptability	The extent of satisfaction with the complexity, comfort, and delivery of digital mental health care through Inuka.	● General population’s degree of comfort in receiving mental health care through Inuka. ● The quality of the session and user satisfaction are automatically measured inside the app. After every session, users are asked to rate the session’s quality on a scale of 0 to 5 stars (commonly used in online and app settings). ● The perceptions of lay counselors toward providing mental health services to the general population digitally. ● The perceived complexity of digital mental health provision (Inuka) by administrators and lay counselors.	● Semistructured interviews with the general population, lay counselors, and administrators ● MAUQ scores [25]	Preimplementation and postimplementation
Appropriateness	The perceived fit, relevance, or compatibility of digital mental services in the Zimbabwean context to address the huge burden of common mental health disorders in the Zimbabwean general population.	● Is Inuka consummate with lay counselors’ roles or job expectations? ● Is accessing mental services through Inuka appropriate given the differences in technology familiarity and access by the Zimbabwean general population?	● Semistructured interviews with the general population ● Semistructured interviews or focus group discussions with administrators ● MAUQ scores [25]	Preimplementation and postimplementation
Fidelity	The extent to which the Inuka intervention (ie, problem-solving approach) protocol is adhered to.	● Are the same procedures being followed in providing mental health services through Inuka? ● Is the treatment algorithm being followed as per the protocol?	● Semistructured interviews with lay counselors, administrators, and supervisors ● Interviews with the general population ● Review of session transcripts ● Review of lay counselors’ ratings	Midimplementation and postimplementation

#### Mental Health Outcomes

##### Primary Clinical Outcomes 

A reduction in CMDs was the primary clinical outcome, and this was measured using the SSQ [14], Patient Health Questionnaire-9 (PHQ-9) [26], and Generalized Anxiety Disorder-7 scale (GAD-7) [27]. All outcome measures have been validated and used extensively in Zimbabwe. The SSQ is a binary (yes/no), 14-item, native Zimbabwean anxiety and depression screener [14]. Scores range from 0 to 14, with scores ≥9 regarded as indicating psychiatric morbidity. The PHQ-9 (9 items) and GAD-7 (7 items) are generic depression and anxiety screeners, respectively [27,28]. On both scales, respondents rate the frequency of the experience of enlisted depressive or anxiety symptoms in the previous 2 weeks on a 4-point Likert scale, ranging from “not at all” (0) to “all the time” (3) to give a cumulative score ranging from 0 to 27 and 0 to 21 for the PHQ-9 and GAD-7, respectively. PHQ-9 scores of 0-9, 15-19, and 20-27 are considered to indicate minor, moderate, and severe depression, respectively [26]. For GAD-7, the criteria values for low, moderate, and severe anxiety are 5, 10, and 15, respectively [27].

##### Secondary Clinical Outcomes

Health-related quality of life **(**HRQoL), disability and functioning, and social support were the secondary outcomes, and these were measured using the EuroQol-5 dimensions (EQ-5D), WHO Disability Assessment Schedule (WHODAS), and multidimensional scale of perceived social support (MSPSS), respectively. The EQ-5D is a generic self-report HRQoL outcome measure [29]. Respondents rate challenges with mobility, self-care, usual activities, pain/discomfort, and anxiety/depression on a 3-point scale. Participants also rate their health on a visual analog scale that ranges from 0 to 100, with higher scores indicating higher perceived HRQoL. Normative utility scores are available for the Zimbabwean population [29]. The WHODAS 2.0 is a brief (12 items) and extensively used generic disability and function outcome measure [30]. It consists of 6 domains (ie, cognition, mobility, self-care, socialization ability, life activities, and community participation), with higher scores indicating greater disability [30]. The MSPSS is an extensively used and psychometrically robust social support outcome measure. Social support sources are categorized as family, friends, and significant others, with 4 items per domain [31]. The scores range from 12 to 36, with higher scores indicating greater social support [32].

### Sample Size

Although formal sample size calculations are not obligatory for pilot studies [33], we sought to recruit 102 participants. Based on the Friendship Bench intervention efficacy evaluation study [14], assuming a 6.7 reduction in SSQ scores after the intervention (µ_0_=10.5, SD 1.4 and µ_1_=3.8, 95% CI 3.3-4.3), the expected minimum number of cases per group was 51 at 95% CI, 90% goal power (β), and anticipated 9% loss to follow-up.

### Analytical Methods

We used descriptive statistics to report the sociodemographic characteristics of the study population. The Shapiro-Wilks test was used to test for the normality of all data before using either parametric tests (eg, *t* tests) or nonparametric tests (eg, Mann-Whitney *U* test). We used the chi-squared test for binary outcomes and the *t* test for continuous outcomes. For all tests, the level was set at α≤.005. Quantitative analyses were performed using SPSS (Version 25.2; IBM Corp) and Stata (Version 16; StataCorp). Implementation parameters were assessed at the cluster and individual levels using mixed methods. Acceptability and appropriateness were assessed preimplementation and postimplementation. Feasibility, adoption/uptake, and fidelity were assessed postimplementation (Table 1). Qualitative data were analyzed using an inductive thematic analysis with the CFIR as a reference frame [24].

### Ethical Considerations

The study was approved by the Medical Research Council of Zimbabwe (MRCZ/A/2566). Participants were treated as autonomous agents and participated in the study voluntarily. Participants either gave written or verbal consent before participating in the study. All participants were assigned a numeric code, and data were deidentified before analysis. Only the PI and technical support staff had access to the database with identifiable information that included contact numbers and email addresses. Moreover, participants were appropriately referred for further clinical management whenever necessary. Cases reporting suicidal ideation or hallucinations were immediately referred for further evaluation and treatment by clinical psychologists and psychiatrists.

## Results

### Participant Flowchart

**Figure 1:**
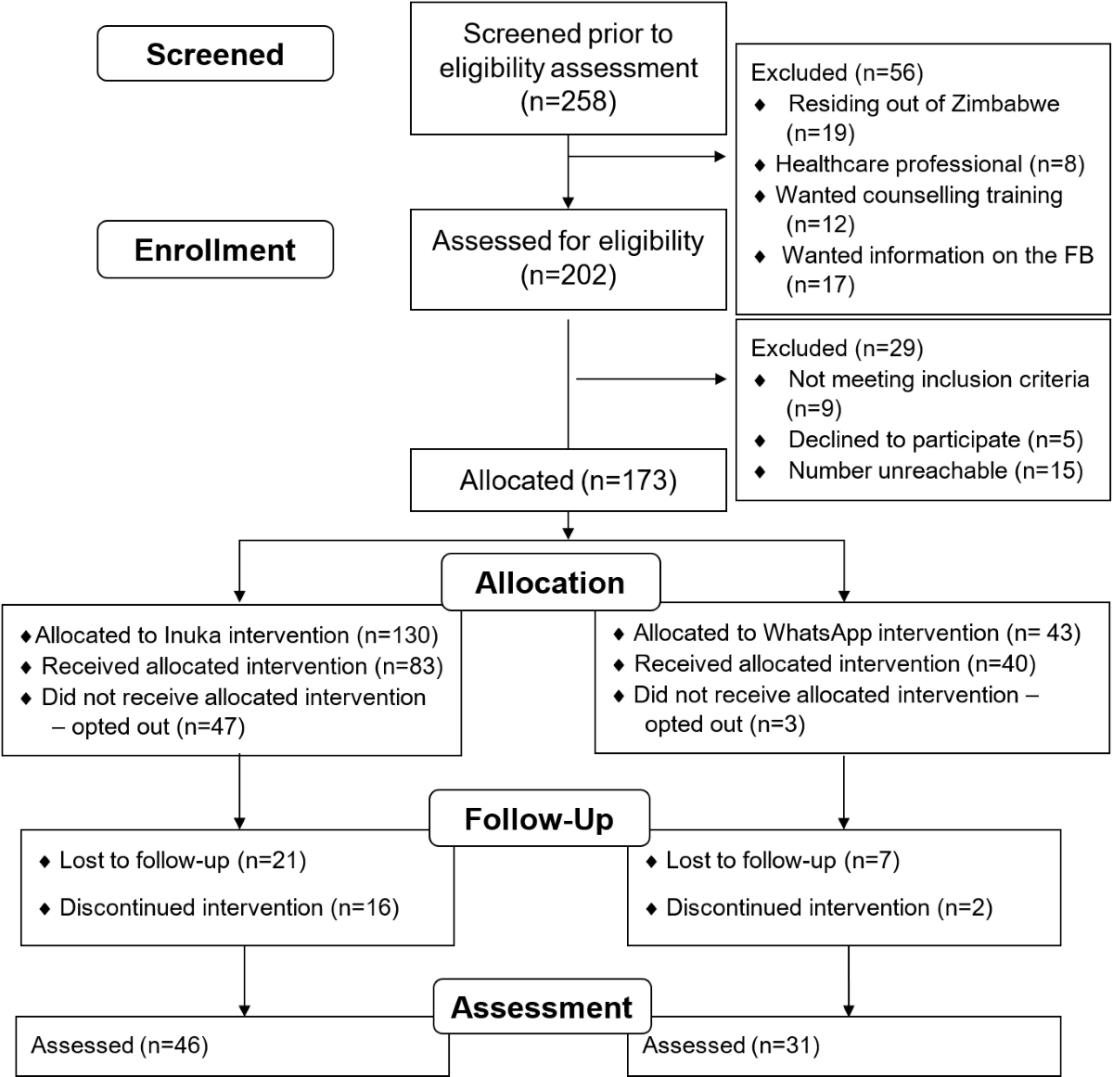
Participants' characteristics.

Figure 1 shows the participant flowchart, that is, the number of participants screened, enrolled, allocated to interventions, lost to follow-up, and analyzed. Altogether, 258 participants were screened over 6 months, with 202 assessed for eligibility, and 173 people who met the inclusion criteria were assigned to either the experimental arm (n=130) or the control arm (n=43), resulting in the recruitment of 29 participants per month (Figure 1). Eventually, 76 participants were assessed.

### Participant Characteristics

As seen in Table 2, the mean age of the participants was 24.4 (SD 5.3) years. Most participants were female (50/76, 66%), were enrolled in or had completed tertiary education (42/76, 55%), never married (53/76, 70%), were unemployed/students (49/76, 64%), and reported financial inadequacy (33/76, 43%) and food insecurity (32/76, 42%). Moreover, most participants had not been hospitalized in the past month (65/76, 86%) and did not experience an adverse event (42/76, 55%). The mean daily smartphone usage was 8 (SD 3.5) hours. Smartphones were mainly used for social media (75/76, 99%), communication (74/76, 97%), and academic work (56/76, 74%). Overall, the groups were comparable at baseline, except for employment status (χ^2^_3_=7.80; *P*=.05). There were more students in the WhatsApp group than in the Inuka group (17/31, 55% vs 11/45, 24%).

**Table 2:** Participant characteristics (N=76).

Variable			Inuka group (n=45)		WhatsApp group (n=31)		Total (N=76)		Statistic, χ^2^ or *t* value (df)		*P* value
**Gender, n (%)**									0.296^a,b^ (1)		.59
	Female	28 (62)		22 (71)		50 (66)					
	Male	17 (38)		9 (29)		26 (34)					
Age, mean (SD)			25.2 (6.0)		23.2 (4.0)		24 (5)		1.629^c^ (74)		.11
**Educational level, n (%)**									0.587^a,b^ (1)		.44
	Secondary	18 (40)		16 (52)		34 (45)					
	Tertiary	27 (60)		15 (48)		42 (55)					
**Marital status, n (%)**									1.762^a^ (2)		.41
	Currently married	8 (18)		5 (16)		13 (17)					
	Never married	33 (73)		20 (65)		53 (70)					
	Other	4 (9)		6 (19)		10 (13)					
**Employment status, n (%)**									7.799^a^ (3)		.05
	Formal	9 (20)		5 (16)		14 (18)					
	Informal	9 (20)		4 (13)		13 (17)					
	Student	11 (24)		17 (55)		28 (37)					
	Unemployed	16 (36)		5 (16)		21 (28)					
**Financial adequacy, n (%)**									1.646 (4)^a^		.88
	Very inadequate	2 (4)		0 (0)		2 (3)					
	Inadequate	18 (40)		13 (42)		31 (41)					
	Somewhat adequate	17 (38)		11 (36)		28 (37)					
	Adequate	7 (16)		6 (19)		13 (17)					
	Very adequate	1 (2)		1 (3)		2 (3)					
**Food security, n (%)**									3.984^a^ (3)		.26
	Inadequate	3 (7)		5 (16)		8 (11)					
	Somewhat adequate	13 (29)		11 (36)		24 (32)					
	Adequate	19 (42)		7 (23)		26 (34)					
	Very adequate	10 (22)		8 (26)		18 (24)					
**Hospital admission, n (%)**									0.000^a,b^ (1)		.99
	No	39 (87)		26 (84)		65 (86)					
	Yes	6 (13)		5 (16)		11 (15)					
**Adverse event, n (%)**									0.088^a,b^ (1)		.77
	No	26 (58)		16 (52)		42 (55)					
	Yes	19 (42)		15 (48)		34 (45)					
Smartphone usage in hours, mean (SD)			8.7 (4.0)		8.8 (2.6)		8.8 (3.5)		−0.130^c^ (74)		.90
**Main smartphone usage function, n (%)**									1.027^a^ (3)		.80
	Social media	44 (98)		31 (100)		75 (99)					
	Academic work	37 (82)		19 (61)		56 (74)					
	Communication	43 (96)		31 (100)		74 (97)					
	Other	10 (22)		7 (23)		17 (22)					

^a^χ^2^ value.

^b^With Yates’ correction of continuity.

^c^*t* value.

### Inuka Session Analysis

Table 3 summarizes the number of completed sessions, cancellation rate, and reasons for incomplete sessions. Eighty-three users signed up and completed at least one session. The average completion rate was 3 sessions per participant. Among the scheduled sessions, the nonattendance proportions for participants and lay counselors were 22.4% (95/424) and 5.9% (25/424), respectively, and 9.2% (39/424) of sessions were cancelled.

**Table 3:** Inuka session overview.

Session status	Value (N=424), n (%)
The user did not show up	95 (22.4)
Session completed	240 (56.6)
The lay counselor did not show up	25 (5.9)
Session cancelled	39 (9.2)
No one showed up for the session	25 (5.9)

### Qualitative Implementation Outcomes

Below are the key findings from the qualitative analysis of the implementation outcomes. The subthemes and additional vivid verbatim quotes are presented in Multimedia Appendix 2.

#### Acceptability

Both users and lay counselors revealed that Inuka was an acceptable model for mental health provision. The ability to access mental health care remotely was the most salient feature. Remote access was highly valued as it enabled access to mental health care during the COVID-19 total lockdowns, with a lay counselor saying the following:

In the wake of the COVID-19 restrictions; the Inuka app makes it very easy to connect with clients since we are not able to conduct physical sessions.

Counselor #2

Moreover, the double anonymity offered by the Inuka app and data security were viewed as vital features. Anonymity increased accessibility to mental health care services and increased patients’ ability to open up to their therapists. One patient made the following statement:

…it is enjoyable and helpful at once…the hiding of identity part is brilliant and encouraging… [Patient #7]

#### Feasibility

Users and lay counselors pointed out that Inuka could be successfully used to provide mental health services in Zimbabwe. One patient commented as follows:

The application is very easy to use…

Patient #1

A lay counselor made the following statement:

It was very easy to use the app… It took me three days to learn how to use the app.

Counselor #5

However, the app users raised concerns about the app’s stability, which affected usability. Challenges in connectivity were reported as a potential impediment to digital mental health care services in the Zimbabwean context. One lay counselor made the following statement:

At times, it is difficult to complete sessions due to connectivity challenges…

Connectivity challenges negatively affected some of the clients’ experiences. Further, the app’s responsiveness and stability were also seen as areas of improvement. Technical glitches were prevalent, and these negatively affected therapy progression. Patients said the following:

My counselor's network was bad…she was late replying always, and I ended up losing focus…

Patient #2

The app was laggy and disconnected frequently…

Patient #7

#### Appropriateness

Lay counselors expressed that the Inuka app was congruent with their roles or job expectations, with the app offering a structured way of delivering services. Clients expressed the ability to connect or form a therapeutic alliance with their counselors over the digital sessions. One patient made the following statement:

Yeah, it was great. I felt like I was talking to a real friend...

Patient #5

#### Adoption/Uptake

Both administrators and lay counselors concurred that Inuka could be integrated within the Friendship Bench to complement physical sessions. The ubiquity of digital innovations was viewed as a low-hanging fruit to increase mental health coverage in low-resource settings. One lay counselor made the following statement:

…digital mental health innovations are the future…we definitely need to embrace changes in times…Inuka offers that…

Counselor #7

However, technical glitches, usability (eg, navigation), and connectivity challenges were viewed as potential barriers to integrating Inuka into routine care. One lay counselor revealed challenges in navigation that may negatively affect user experience:

…it was slow in that I struggle to navigate from the action card to the actual chat platform … it took a bit time than it should…

Counselor #5

#### Fidelity

The lead psychologist’s audit checks revealed that lay counselors adhered to the PST protocol. The structured PST steps on the app made it easier for lay counselors to follow through with the sessions with fidelity. A lay counselor made the following statement:

There is no way you can forget all the necessary PST steps as the information is all included in the app…

Counselor #4

### Inuka Usability

#### MAUQ Outcomes

Table 4 shows lay counselors’ usability ratings of Inuka regarding ease of use, esthetics, and usefulness. The lay counselors assigned the highest ratings for ease of use and the lowest ratings for satisfaction and esthetics. The mean MAUQ score was 78.5 (SD 13.2), denoting high perceived usability. The frequencies of responses on the MAUQ are presented in Multimedia Appendix 3.

**Table 4:** Lay counselors’ mHealth App Usability Questionnaire scores (N=8).

mHealth App Usability Questionnaire (MAUQ) variable	Mean score (SD)	Median score (Q1-Q3)	Score range (min-max)
Ease of use and satisfaction (40 points)	27.5 (6.2)	29.0 (24.0-31.5)	19.0 (16-35)
System information arrangement (30 points)	25.1 (3.4)	24.0 (24.0-25.0)	11.0 (22-33)
Usefulness (35 points)	25.9 (5.1)	27.0 (22.5-29.0)	16.0 (17-33)
MAUQ total score (105 points)	78.5 (13.2)	80.5 (70.0-83.5)	42.0 (59-101)

#### In-App Ratings

Participants were satisfied with the Inuka app. The mean app and session ratings were 4.2 (SD 1.2) and 4.5 (SD 1.1), respectively. The descriptive statistics are displayed in Table 5.

**Table 5:** Clients’ in-app ratings (N=83).

Rating	Mean value (SD)	Range (min-max)	Median value (Q1-Q3)
App rating	4.2 (1.2)	4 (1-5)	5 (3-5)
Session rating	4.5 (1.1)	4 (1-5)	5 (4-5)

### Mental Health Outcomes

Table 6 compares the mental health outcomes of the 2 groups at baseline and follow-up. The table shows changes in depression, anxiety, disability and functioning, social support, and HRQoL over the follow-up period. Both groups were comparable at baseline, except for depression scores. PHQ-9 scores were higher in the WhatsApp group than in the Inuka group (t_74_=−2.725; *P*=.008). Generally, there were declines in depression, anxiety, and disability and functioning, and corresponding increases in social support and HRQoL in both groups (Table 6). A 1-way ANOVA was performed to test for changes in the mean PHQ-9 scores at baseline and follow-up. It yielded statistically significant differences between the 2 groups (*F*_2,73_=7.67; *P*<.001), with the WhatsApp group exhibiting a great decline in depression scores. A 1-way ANOVA was also performed to test for changes in the mean EQ-5D visual analog scale scores at baseline and follow-up. It yielded a statistically significant difference between the 2 groups (*F*_2,73_=7.287; *P*=.001), with the Inuka group exhibiting great gains in HRQoL scores.

**Table 6:** Mental health outcomes (N=76).

Construct- outcome measure	Baseline					Follow-up		Between-group comparisons		
	Inuka group (n=45), mean (SD)	WhatsApp group (n=31), mean (SD)	*t* (df)	*P* value	Inuka group (n=45), mean (SD)		WhatsApp group (n=31), mean (SD)		*F* (df)	*P* value
Common mental disorders (Shona Symptoms Questionnaire)	8.0 (2.6)	8.1 (2.0)	10.116 (74)	.91	5.4 (2.1)		6.48 (1.6)		2.63 (2,73)	.08
Depression (Patient Health Questionnaire-9)	10.4 (5.5)	13.2 (2.3)	−2.725 (74)	.008	6.9 (4.0)		9.7 (2.8)		7.67 (2,73)	<.001
Anxiety (Generalized Anxiety Disorder-7)	10.5 (5.8)	12.1 (2.7)	−1.630 (74)	.15	7.2 (4.4)		9.1 (2.7)		2.95 (2,73)	.06
Disability and functioning (WHO^a^ Disability Assessment Schedule)	23.1 (9.0)	22.1 (5.4)	−1.766 (74)	.08	18.4 (5.1)		17.8 (2.1)		0.273 (2,73)	.76
Social support (multidimensional scale of perceived social support)	25.9 (5.2)	27.7 (3.1)	−1.766 (74)	.08	28.2 (4.5)		28.4 (3.3)		1.545 (2,73)	.22
HRQoL^b^ (EQ-5D^c^ utility score)	0.807 (0.139)	0.745 (1.69)	1.735 (74)	.09	0.859 (0.124)		0.850 (0.077)		1.49 (2,73)	.23
HRQoL (EQ-5D visual analog scale score)	62.1 (20.2)	58.6 (12.5)	0.870 (74)	.39	73.2 (11.5)		63.6 (10.4)		7.287 (2,73)	.001

^a^WHO: World Health Organization.

^b^HRQoL: health-related quality of life.

^c^EQ-5D: EuroQol-5 dimensions.

### Data Dissemination

We will communicate the study findings to the health care professionals and relevant groups associated with the study through policy briefs, and oral and conference presentations. As for the participants, we will summarize the study findings in simplified language, and information will be disseminated using leaflets with summarized findings.

## Discussion

### General Findings

This study used a pragmatic nonequivalent control group quasiexperimental design to explore the feasibility of running a future RCT. The study also examined the feasibility, acceptability, and preliminary clinical effectiveness of Inuka compared with the Friendship Bench intervention delivered via WhatsApp. Overall, study outcomes showed that it is feasible to run a future large-scale RCT and lend support to the feasibility and acceptability of Inuka, including evidence of preliminary effectiveness. However, connectivity and usability challenges are potential impediments to the scale-up of Inuka.

### Feasibility of a Future Clinical Trial

An optimal recruitment rate demonstrated the feasibility of a future RCT. We screened 258 participants over 6 months (43 participants per month). The recruitment rate is comparable to that in an almost similar local study [34]. A pilot trial exploring the feasibility and acceptability of a task-shifted PST intervention to improve adherence in HIV care in Harare, Zimbabwe, yielded a recruitment rate of 27 participants per month [34]. The high recruitment rate may be attributable to the increased awareness and acceptability of mHealth interventions in the study setting [35-37]. The ongoing COVID-19 pandemic has brought the importance of mental health to the forefront [38]. COVID-19 containment measures, such as lockdowns, have resulted in an exponential increase in mental health issues; hence, there is an impetus to increase service coverage [38,39]. Another possible reason for the increased uptake could be the campaign of the Zimbabwean Ministry of Health and Child Care and the WHO to increase the awareness and coverage of mental health services in Zimbabwe [40].

The high recruitment rate could be credited to our multimodal dissemination strategy. We publicized the pilot study through social media, the Friendship Bench website [23], and radio channels. The study sensitization was engrafted in the Friendship Bench’s broader community engagement strategy to increase awareness of the mental health effects of the COVID-19 pandemic, including signposting clients to care [35]. The COVID-19 pandemic has led to an exponential increase in social media usage, and this coupled with high mobile penetration [37,39] increased our social media reach.

We had initially planned to randomize clients to interventions; however, this was impractical. Most clients meeting the inclusion criteria preferred Inuka (n=130) to WhatsApp (n=43). The double anonymity offered by the app was the major pull factor, and this is consistent with other studies [13]. Anonymity decreases stigmatization, which enhances the uptake of mental health care services [13,41]. Unfortunately, there was a high loss to follow-up in the Inuka group. Around 36.1% of clients in the Inuka group did not receive the allocated intervention, while this rate was 7.0% in the WhatsApp group. Connectivity challenges were cited as the main barriers to accessing the Inuka intervention. Poor connectivity and high data costs are salient barriers to the scale up of mHealth innovations in low-resource settings [36,37]. There is a need to iteratively improve the Inuka platform to a data-lite version. Methodologically, future RCTs should consider a large sample and block randomization enrollment, with 5 participants randomized to the Inuka group for every participant assigned to the control group. Moreover, consideration may be made to compare Inuka to the in-person Friendship Bench intervention. This was impossible owing to the ongoing COVID-19 pandemic. Overall, the loss to follow-up for both groups was 25.3%, which is comparable to a similar study evaluating the feasibility of using lay counselors for improving HIV care adherence [34]. Loss to follow-up is inevitable; however, we employed several retention strategies, including texting reminders and encouraging clients to activate in-app reminders. The Inuka app sends reminders to both lay counselors and clients an hour and 30 minutes before the session. Moreover, upon signing up, the system sends an automated confirmatory email with a calendar file that can be added to the client’s digital calendar. However, clients rarely used this feature, necessitating multiple retention strategies such as reminder text messages and phone calls. Taken together, this pilot study shows the feasibility of a future trial. However, there need to be considerations for the allocation mechanism and efforts to increase adherence to scheduled sessions.

### Feasibility and Acceptability of Inuka

This pilot study also demonstrated the feasibility and acceptability of implementing Inuka, a digital mental health intervention in Zimbabwe, a low-resource country. The ongoing COVID-19 pandemic has increased the utility of digital mental health interventions [39]. To the best of our knowledge, this is the first study to explore the utility of digital mental health solutions in Zimbabwe and one of the few studies in Africa [18,36,39]. Digital mental health solutions can alleviate access barriers, including destigmatizing access to mental health care and offering convenience compared with physical sessions [12,42]. For example, the double anonymity offered by Inuka was highly valued by both participants and lay counselors. However, connectivity and usability challenges are potential impediments to the feasibility of mHealth interventions in low-income settings [42]. The beta version used during the pilot required a much more stable internet connection than WhatsApp. Internet speeds in Zimbabwe depend much on geographical location. For example, connection speeds are faster and more stable in low-density areas than in high-density areas, with rural areas having poorer connectivity than urban areas [40]. Such disparity affects the feasibility of an equitable roll out of mHealth interventions in low-resource settings, necessitating the exploration of reasonable alternatives. During the early phases of the study, we received several complaints from both clients and lay counselors about the app’s connectivity and stability. The developers reconfigured the app by changing to an Angular framework that is data lite and is compatible with unreliable internet speeds in low-resource settings such as Zimbabwe. Moreover, users suggested that offline access to some key features (eg, chat user interface), voice calls/messages, and push notifications of new messages could improve the utility of Inuka further. The piloting phase indicated a stern need to elicit constant feedback and troubleshooting to optimize mHealth service delivery [42,43].

Despite the predictable challenges with mHealth technologies, both lay counselors and clients were highly satisfied with the Inuka app. The satisfaction rate is comparable to that in other studies and is vital for enhancing adherence and improving treatment effectiveness and efficiency [9,10,13,17]. As in other settings [13], our preliminary outcomes suggest that Inuka can offer PST sessions with increased fidelity as it offers lay counselors essential reminders of all critical steps. Supervisors consisting of experienced clinical psychologists and psychiatrists could remotely access the session transcripts. The ability to remotely access transcripts is time-saving, increases user satisfaction and quality control, and is a salient feature of mHealth technologies compared with in-person sessions [13]. Moreover, the Inuka platform enables real-time tracking of session attendance and changes in client well-being scores, and enables clients to submit anonymized feedback on the quality of the service received. These salient features collectively enhance quality control, including strengthening the clinical supervision process, which is essential for optimal treatment outcomes.

### Preliminary Clinical Effectiveness

Although our study was not powered to demonstrate clinical effectiveness, study outcomes provided evidence of preliminary effectiveness. Participants showed improvements in anxiety and depression, and increased functioning and HRQoL. Systematic reviews have demonstrated that mHealth apps are clinically effective as face-to-face therapies [13,41]. Inuka can be an alternative or complimentary delivery model to the original in-person Friendship Bench intervention to improve mental health outcomes due to the relative advantage of enabling greater access to care. Across both arms, lay counselors provided counseling, which is a more sustainable care provision in low-resource settings that lack human resources and capital investment in mental health care [14]. However, the lack of in-person contact between a client and coach is a potential drawback for therapeutic alliance in mHealth interventions [44]. There is a greater need to explore ways to optimize client experiences to increase the utility and engagement of mHealth technologies [13,41]. Optimization of mHealth technologies is crucial in low-income countries where mHealth technologies could help close the sizeable mental health care gap [12].

### Limitations and Strengths

Several methodological limitations warrant a cautious interpretation of the findings. First, participants were conveniently assigned to intervention groups, which may have introduced selection bias. Randomization was impractical given the differences in smartphones and high-speed stable internet access among participants. Second, there was a considerable loss to follow-up in the Inuka intervention arm. Network changes, power outages, and forgetting were the primary reasons for nonattendance. Beyond the pilot study, the Inuka team has iteratively reconfigured the app to a data-lite Angular framework, including WhatsApp integration. The iterations will increase coverage and mitigate connectivity challenges. The new app requires rigorous testing before scaling up. Moreover, future studies should explore the effectiveness of implementation strategies (eg, text reminders) to enhance adherence to scheduled sessions. A strength of this study was that lay counselors successfully implemented the intervention. Task-shifting is a cost-effective mental health care service delivery model [14]. Moreover, the Inuka app enables full access to session transcripts for fidelity assessment, and all interventionists highly adhered to the PST steps.

### Conclusion

The findings of our study suggest that the Inuka intervention is feasible and acceptable in the Zimbabwean context. The high perceived ease of use and satisfaction by clients and lay counselors, the comparable level of uptake of the Inuka intervention when compared with the in-person Friendship Bench intervention, the positive perceptions around the use of technology in mental health care, and the desirability of the anonymity provided by the intervention/app increase the app’s utility. Our findings also provide preliminary evidence of the clinical effectiveness of the Inuka intervention. However, significant barriers to implementation were identified, primarily concerning internet connectivity and mobile app stability. There is a need for iterative app upgrades to increase usability, which will, in turn, improve the scaling up of the mHealth solution. The Friendship Bench and other clinicians should consider using mHealth solutions, such as Inuka, to complement in-person therapies to close the mental health care gap in low-resource settings. Future research should use our findings to optimize the intervention and investigate the clinical implementation and cost-effectiveness of the Inuka intervention using a hybrid RCT design.

## Appendix

Multimedia Appendix 1 Description of the Inuka app.

Multimedia Appendix 2 Qualitative analysis of implementation outcomes.

Multimedia Appendix 3 Mobile App Rating Scale responses.

## Abbreviations

CFIR: Consolidated Framework for Implementation Research
CMD: common mental health disorder
EQ-5D: EuroQol-5 dimensions
GAD-7: Generalized Anxiety Disorder-7
HRQoL: health-related quality of life
MAUQ: mHealth App Usability Questionnaire
mHealth: mobile health
MSPSS: multidimensional scale of perceived social support
PHQ-9: Patient Health Questionnaire-9
PI: principal investigator
PST: problem-solving therapy
RA: research assistant
RCT: randomized clinical trial
SSQ: Shona Symptoms Questionnaire
WHO: World Health Organization
WHODAS: World Health Organization Disability Assessment Schedule
