# Supplementary material for: A Digital Mental Health Intervention (Inuka) for Common Mental Health Disorders in Zimbabwean Adults in Response to the COVID-19 Pandemic: Feasibility and Acceptability Pilot Study
Source: JMIR Ment Health. 2022 Oct 7;9(10):e37968. doi: 10.2196/37968 (PMC9555820; doi:10.2196/37968)
Supplement: Multimedia Appendix 1 [file mental_v9i10e37968_app1.pptx]

## Slide 1
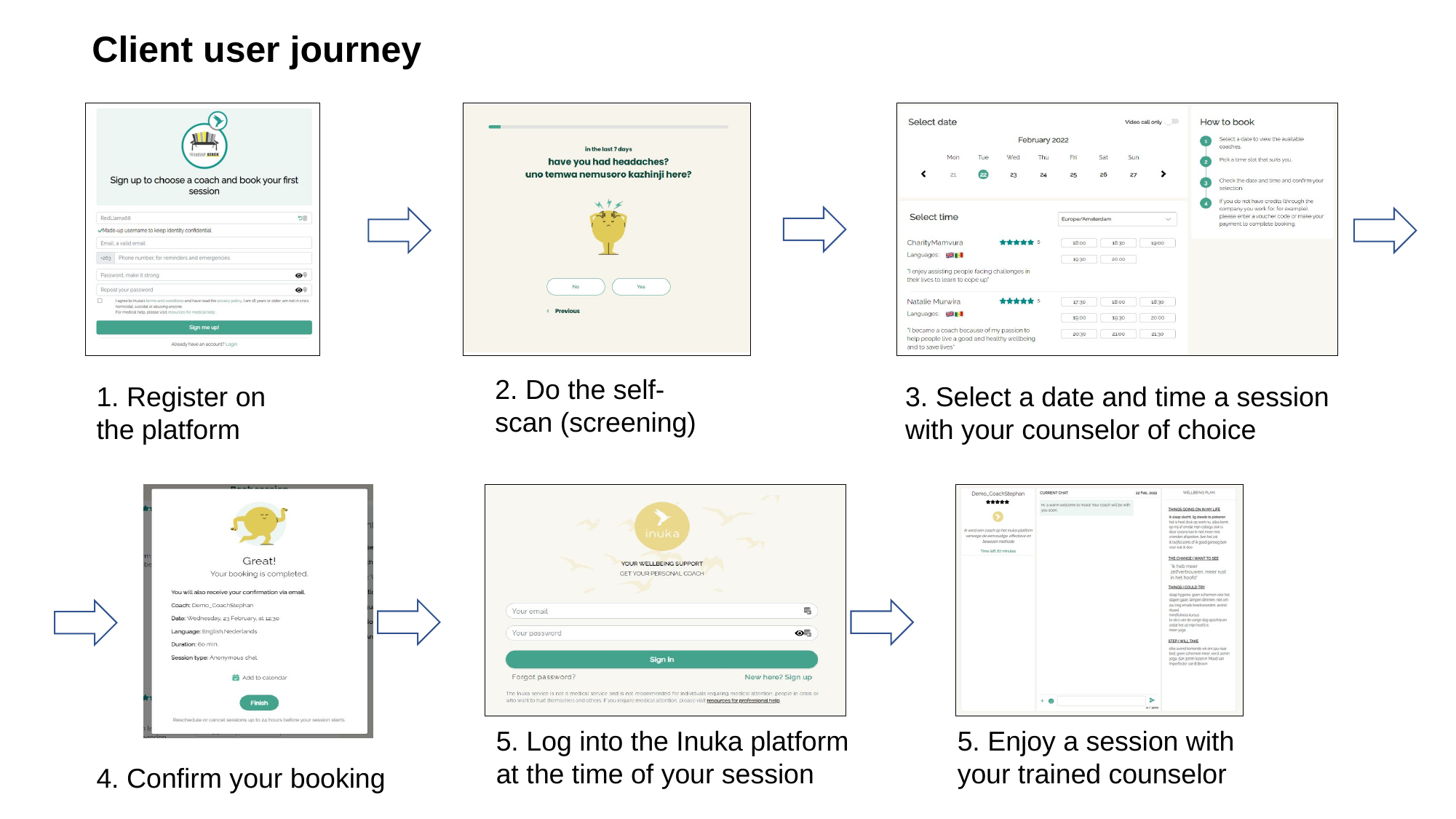

Client user journey
2. Do the self-scan (screening)
3. Select a date and time a session with your counselor of choice
1. Register on the platform
4. Confirm your booking
5. Log into the Inuka platform at the time of your session
5. Enjoy a session with your trained counselor

## Slide 2
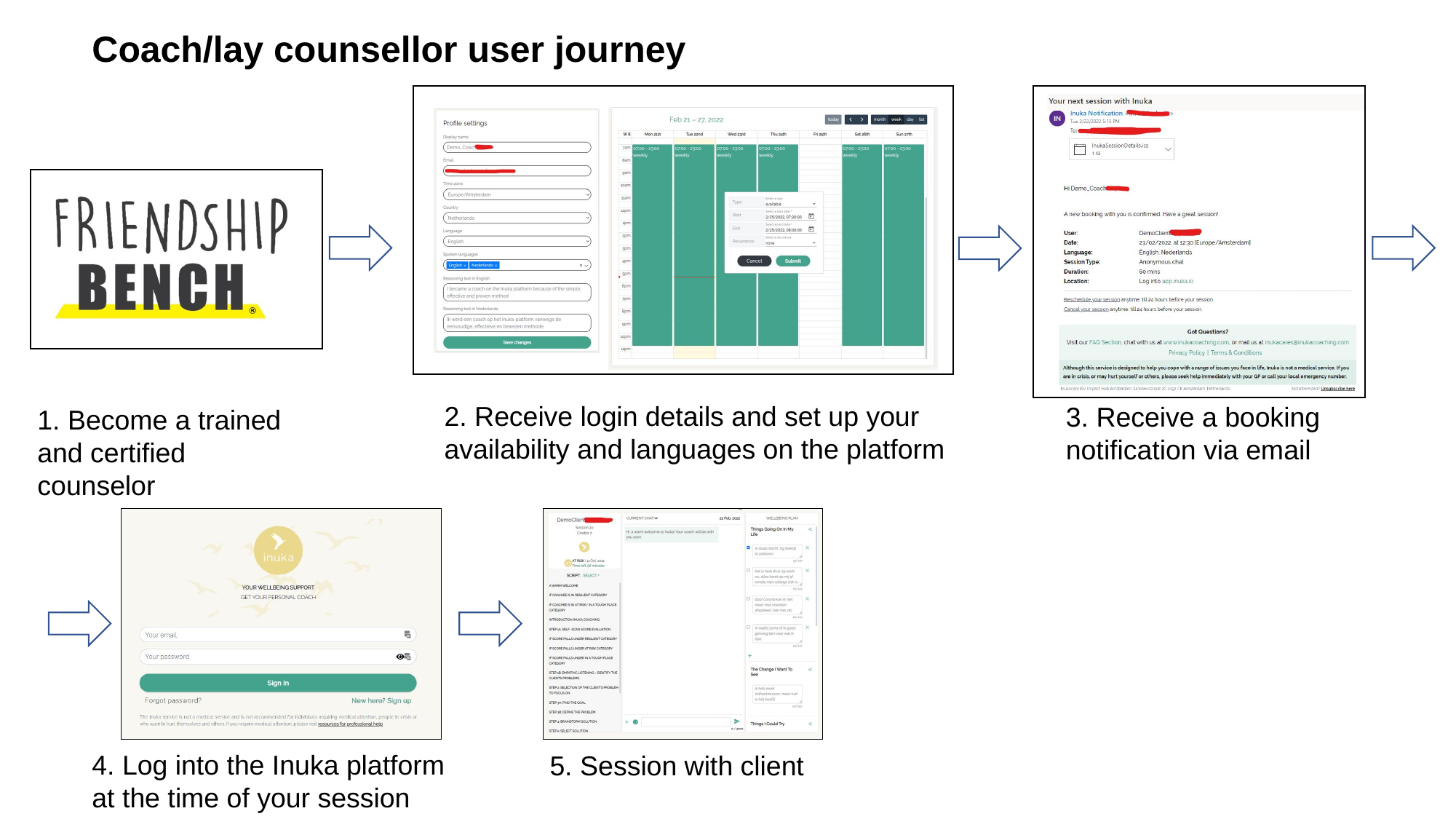

Coach/lay counsellor user journey
2. Receive login details and set up your availability and languages on the platform
3. Receive a booking notification via email
1. Become a trained and certified counselor
4. Log into the Inuka platform at the time of your session
5. Session with client

## Slide 3
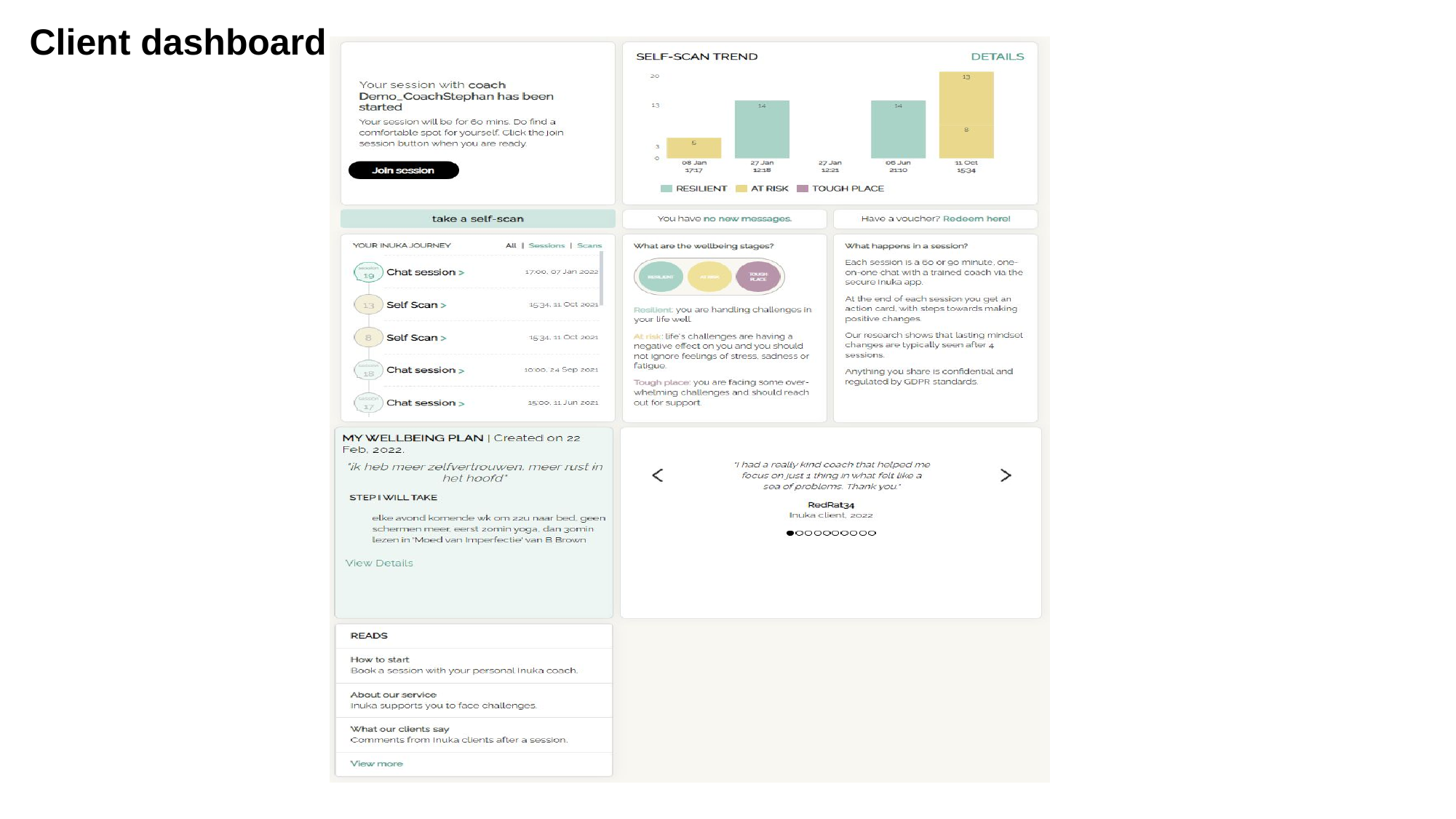

Client dashboard

## Slide 4
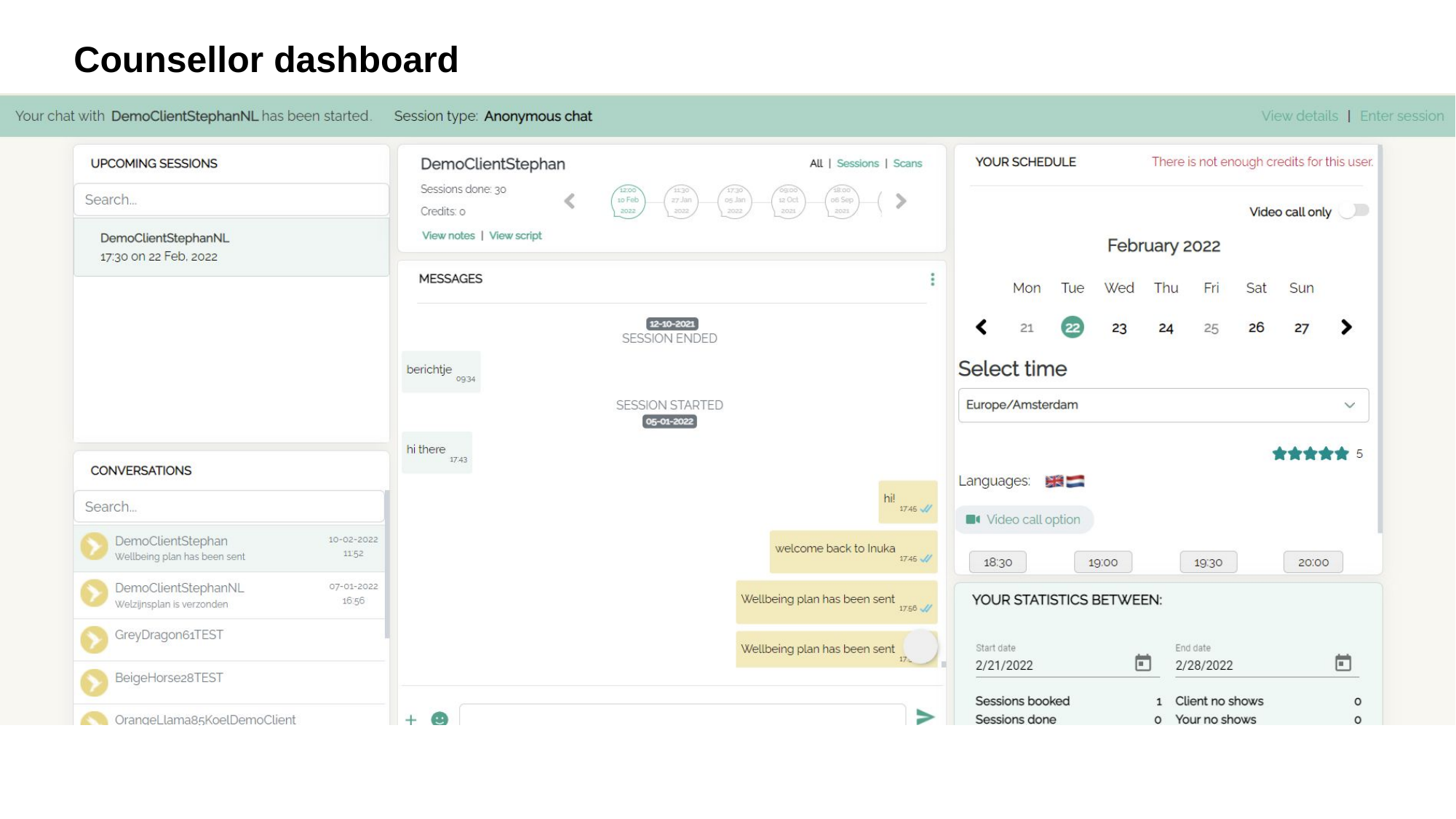

Counsellor dashboard

## Slide 5
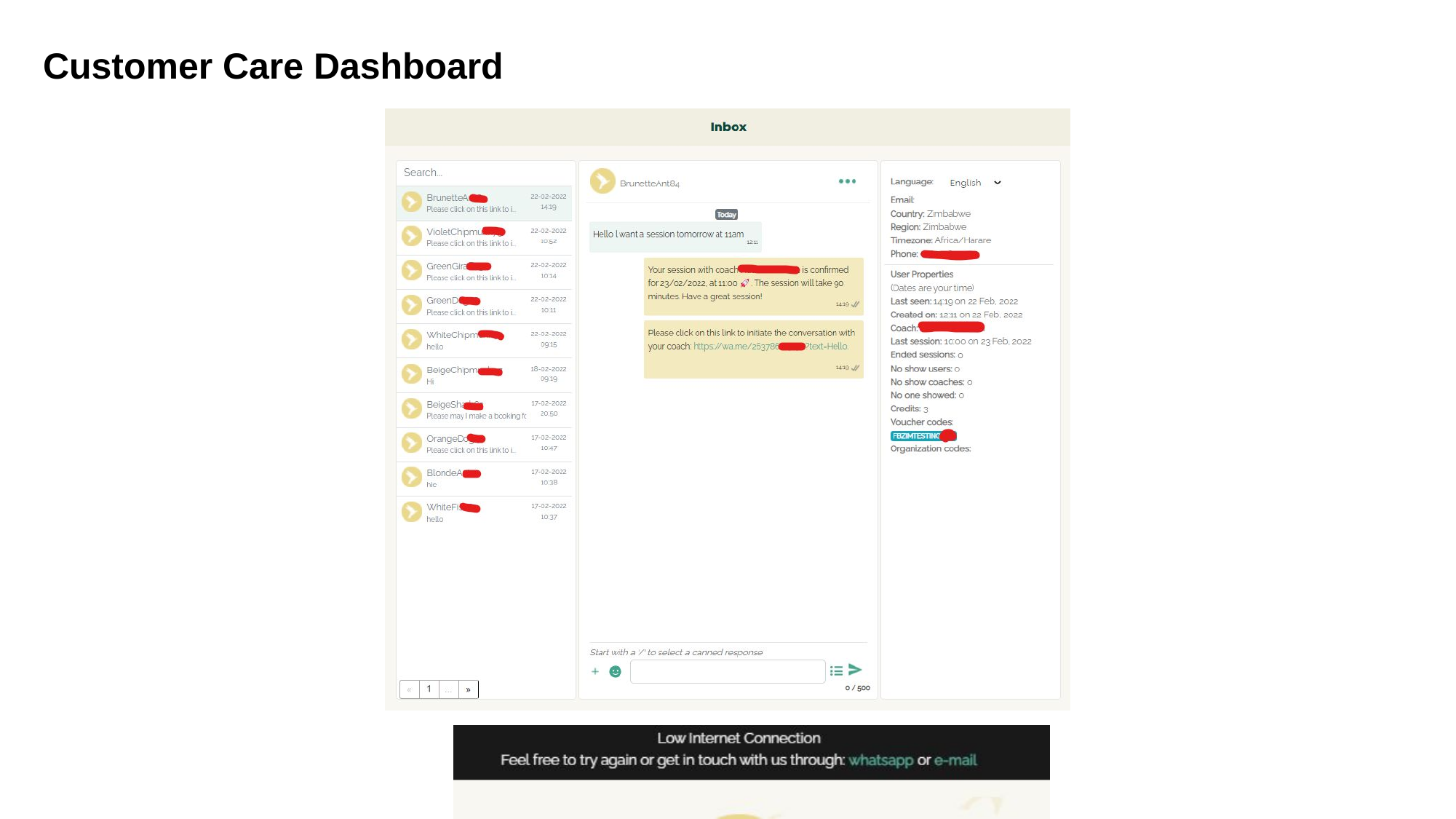

Customer Care Dashboard
