# Supplementary material for: A Digital Mental Health Intervention (Inuka) for Common Mental Health Disorders in Zimbabwean Adults in Response to the COVID-19 Pandemic: Feasibility and Acceptability Pilot Study
Source: JMIR Ment Health. 2022 Oct 7;9(10):e37968. doi: 10.2196/37968 (PMC9555820; doi:10.2196/37968)
Supplement: Multimedia Appendix 2 [file mental_v9i10e37968_app2.docx]

| Implementation Outcome | Sub-theme(s) | Verbatim quotes | Respondent |
| --- | --- | --- | --- |
| Acceptability | Convenience  /Accessibility | “The coach (lay counsellor) is very friendly and will definitely recommend this application and service to my family and friends........ It's an eye-opener -P3”. | Patient |
|  |  | “In the wake of the COVID-19 restrictions; the Inuka app makes it very easy to connect with clients since we are not able to conduct physical sessions - C2”. | Patient |
|  | Privacy/anonymity | “Inuka definitely improves how clients access counselling services ... it makes a huge difference for clients who want their identity to remain unknown…C8”. | Lay counsellor |
|  |  | “I like that the digital app allows the client to fully open up because sometimes with face to face, they may not be as comfortable … C5”. | Lay counsellor |
|  |  | “…the information that you share with your client will remain confidential and isn’t accessible to others, that is very good- C6”. | Lay counsellor |
|  |  | “It’s enjoyable and helping at once ....the hiding of identity part is brilliant and encouraging…**P7**”. | Patient |
|  |  | “The app made it very easy for me to open up… you know at times it is not easy to tell others what you will be going through as you will be judged… I felt so confident as I was assured that my coach would not have my details and that all chats were stored securely ...P6”. | Patient |
| Feasibility | Ease of use | “The application is very easy to use… **P1**” | Patient |
|  | (Facilitator) | “It was very easy to use the app… It took me three days to learn how to use the app- C5”. | Lay counsellor |
|  | App instability and responsiveness (barrier) | “… you at times have to constantly log in and off, and sometimes you don’t see the messages coming from your client, and the ones that you are sending are not being received by the client, there was a lot going on…C1”. | Lay counsellor |
|  |  | “The app froze a lot…P5” | Patient |
|  |  | “The app was laggy and disconnected frequently…P7”. | Patient |
|  | Technical glitches | “There were times when messages would not be delivered … so you would keep trying press enter several times, and by the time that happens the message would have been sent about eight times, and there was no coming back from that so you would have to start apologising to the client…**C6**”. | Lay counsellor |
|  |  | There was quite a lot of technical glitches sometimes it would take out of the session and message don’t sometimes deliver you fail to log in you know, there were a lot of technical glitches that comes with using virtual methods I guess… **C2**”. | Lay counsellor |
|  | Connectivity challenges (barrier) | At times it is difficult to complete sessions due to connectivity challenges, especially if you are using mobile data bundles… at times, I have to go to the Friendship Bench to access stable Wi-Fi for the sessions .... I wish if the app can be configured to use less bandwidth like WhatsApp…C7”. | Lay counsellor |
|  |  | “My counsellor’s network was bad…she was late replying always, and I ended up losing focus… P2”, | Patient |
|  |  | “…my session was good, but the internet stability would help to maintain the pacing of the sessions…**P4**”. |  |
| Appropriateness | Convenience | “I liked it (Inuka app) because I could use it anywhere, the app is installed on my phone as long as the network is okay I can use it while going to work I can use it at home, so it’s a flexible app and also its arranged in such a way, where the responses are there, there are already set out for you, and that very convenient … | Lay counsellor |
|  | Ability to forge alliances | Yeah, it was great felt like was talking to a real friend...**P5**”. | Patient |
| Adoption/uptake | Complimentary delivery platform | “Inuka can certainly be a great compliment to our physical sessions…C8”. | Lay counsellor |
|  |  | “…digital mental health innovations are the future…we definitely need to embrace changes in times…Inuka offers that…C7”. | Lay counsellor |
|  | Technical glitches as barriers | “…the app was slow in that I struggle to navigate from the action card to the actual chat platform … it took a bit time than it should and so I would struggle with navigating like from one yaa…C5”. | Patient |
|  | Need for iterative upgrades | “I strongly agree that Inuka is app would be useful for counselling, however, there is need to improve how the app functions, I think functionality issues aagh… I think it needs to go through a few more phases of development so that its more stable and is a lot faster so provided that happens I think it was a very, very excellent app: **C4**”. | Lay counsellor |
| Fidelity | Structured support | “I found the app very useful as I could easily access all treatment parameters…for instance…I could easily access treatment goals from the previous session…**C2”.** | Lay counsellor |
|  |  | “There is no way you can forget all the necessary PST steps as the information is all included in the app…**C4**”. | Lay counsellor |
