# Supplementary material for: A Digital Mental Health Intervention (Inuka) for Common Mental Health Disorders in Zimbabwean Adults in Response to the COVID-19 Pandemic: Feasibility and Acceptability Pilot Study
Source: JMIR Ment Health. 2022 Oct 7;9(10):e37968. doi: 10.2196/37968 (PMC9555820; doi:10.2196/37968)
Supplement: Multimedia Appendix 3 [file mental_v9i10e37968_app3.docx]

**Multimedia Appendix 3.** Mobile Application Rating Scale responses (N=8).

| **Statements** | **Strongly disagree=1** | **Disagree=2** | **Neutral=3** | **Agree=4** | **Strongly agree=5** |
| --- | --- | --- | --- | --- | --- |
| 1. The app was easy to use. | 2 | - | - | 3 | 3 |
| 1. It was easy for me to learn to use the app | 1 | - | - | 3 | 3 |
| 1. I like the interface of the app. | 1 | - | 2 | 2 | 3 |
| 1. The information in the app was well organised, so I could easily find the information I needed. | 2 | - | 1 | 1 | 4 |
| 1. I feel comfortable using this app in social settings. | 2 | - | 1 | 1 | 4 |
| 1. The amount of time involved in using this app has been fitting for me. | - | - | - | 7 | 1 |
| 1. I would use this app again. | 1 | - | - | 6 | 1 |
| 1. Overall, I am satisfied with this app. | - | - | 3 | 4 | 1 |
| 1. Whenever I made a mistake using the app, I could recover easily and quickly. | - | 2 | 3 | 2 | 1 |
| 1. This mHealth app provides an acceptable way to deliver healthcare services. | 1 | - | - | 2 | 5 |
| 1. The app adequately acknowledged and provided information to let me know the progress of my action. | - | - | - | 5 | 3 |
| 1. The navigation was consistent when moving between screens | 1 | 3 | 2 | 2 | 0 |
| 1. The interface of the app allowed me to use all the functions (such as entering information, responding to reminders, viewing information) offered by the app. | - | - | 3 | 5 | - |
| 1. This app has all the functions and capabilities I expected it to have. | - | 1 | 5 | 1 | 1 |
| 1. The app would be useful for my healthcare practice. | 1 | - | - | 4 | 3 |
| 1. The app improved my access to delivering healthcare services. | 2 | - | - | 3 | 3 |
| 1. The app helped me manage my patients’ health effectively. | - | - | 4 | 3 | 1 |
| 1. The app made it convenient for me to communicate with my patients. | - | - | 3 | 4 | 1 |
| 1. Using the app, I had many more opportunities to interact with my patients. | - | - | 5 | 2 | 1 |
| 1. I felt confident that any information I sent to my patients using the app would be received. | - | 1 | 1 | 5 | 1 |
| 1. I felt comfortable communicating with my patients using the app. | 1 | - | 2 | 3 | 2 |
